# Deficiency in DGCR8-dependent canonical microRNAs causes infertility due to multiple abnormalities during uterine development in mice

**DOI:** 10.1038/srep20242

**Published:** 2016-02-02

**Authors:** Yeon Sun Kim, Hye-Ryun Kim, Hyongbum Kim, Seung Chel Yang, Mira Park, Jung Ah Yoon, Hyunjung J. Lim, Seok-Ho Hong, Francesco J. DeMayo, John P. Lydon, Youngsok Choi, Dong Ryul Lee, Haengseok Song

**Affiliations:** 1 Department of Biomedical Science, CHA University, Seongnam, Gyeonggi, 463-400 Korea; 2 Department of Pharmacology and Brain Korea 21 PLUS Project for Medical Science, Yonsei University College of Medicine, Seoul 120-752, Korea; 3 Fertility Center of CHA Gangnam Medical Center, CHA University, Seoul, Korea; 4 Department of Veterinary Medicine, College of Veterinary Medicine, Konkuk University, Seoul, 143-701 Korea; 5 Department of Internal Medicine, School of Medicine, Stem Cell Institute, Kangwon National University, Chuncheon, Kangwon 200-701, Korea; 6 Department of Molecular and Cellular Biology, Baylor College of Medicine, Houston, Texas, 77030 USA

## Abstract

DGCR8 is an RNA-binding protein that interacts with DROSHA to produce pre-microRNA in the nucleus, while DICER generates not only mature microRNA, but also endogenous small interfering RNAs in the cytoplasm. Here, we produced Dgcr8 conditional knock-out mice using progesterone receptor (PR)-Cre (Dgcr8^d/d^) and demonstrated that canonical microRNAs dependent on the DROSHA-DGCR8 complex are required for uterine development as well as female fertility in mice. Adult Dgcr8^d/d^ females neither underwent regular reproductive cycles nor produced pups, whereas administration of exogenous gonadotropins induced normal ovulation in these mice. Interestingly, immune cells associated with acute inflammation aberrantly infiltrated into reproductive organs of pregnant Dgcr8^d/d^ mice. Regarding uterine development, multiple uterine abnormalities were noticeable at 4 weeks of age when PR is significantly increased, and the severity of these deformities increased over time. Gland formation and myometrial layers were significantly reduced, and the stromal cell compartment did not expand and became atrophic during uterine development in these mice. These results were consistent with aberrantly reduced stromal cell proliferation and completely failed decidualization. Collectively, we suggest that DGCR8-dependent canonical microRNAs are essential for uterine development and physiological processes such as proper immune modulation, reproductive cycle, and steroid hormone responsiveness in mice.

MicroRNAs are single-stranded non-coding RNAs that function as key elements of gene regulatory networks by directing the translational repression or degradation of complementary target mRNAs^1^,^2^. In general, canonical microRNAs are initially produced as the primary microRNA which is recognized by DGCR8, an RNA binding protein, forming the Microprocessor complex with DROSHA, an RNase III-containing enzyme. The Microprocessor complex cleaves the primary microRNA, resulting in stem-loop pre-microRNAs which are then exported from the nucleus to the cytoplasm by EXPORTIN-5 and subsequently processed to mature microRNAs by DICER. In addition to canonical microRNAs, mature microRNAs, such as mirtrons, can be produced via non-canonical pathways^3^,^4^, suggesting that some clusters of microRNAs could be distinctly regulated by the Microprocessor complex and DICER.

Mouse models with conditional deletions of microRNA processing factor(s) have provided evidence for their critical roles in various aspects of mammalian development and stem cell biology^5^,^6^,^7^,^8^. For instance, two independent studies with conditional deletions of Dicer and Dgcr8 with Zp3-Cre (Dicer^flox/flox^;Zp3-Cre and Dgcr8^flox/flox^;Zp3-Cre) clearly demonstrated that microRNAs are globally suppressed in mouse oocytes^6^,^7^. Interestingly, meiotic abnormalities due to defective spindle formation occurred in Dicer^flox/flox^;Zp3-Cre oocytes but not in Dgcr8^flox/flox^;Zp3-Cre oocytes, suggesting that some phenotypes of Dicer deficient mice result from the dysregulation of endogenous small interfering RNAs (endo siRNAs), rather than microRNAs.

To examine the physiological function(s) of microRNAs in female reproductive tracts, the reproductive phenotypes of mice with conditional deletions of Dicer by anti-Mullerian hormone receptor 2 (Amhr2)-Cre (Dicer^flox/flox^;Amhr2^cre/+^) have been analyzed^9^,^10^,^11^. Although the spectrum of phenotypes is diverse, there are multiple shared abnormalities in female reproductive tracts, such as oviductal cysts, and a decreased weight and length of the uterus. Dicer conditional knockout mice by progesterone receptor (PR)-Cre (Dicer^flox/flox^;PR^cre/+^) showed more severe reproductive phenotypes than those observed in Dicer^flox/flox^;Amhr2^cre/+^ mice^12^. These results strongly suggest that spatiotemporal modes of CRE provide diverse reproductive phenotypes that could be affected by microRNAs. To precisely delineate the functions of microRNAs, especially canonical microRNAs, in female reproductive tracts, mouse models with conditional deletion(s) of not only Dicer, but also other gene(s) involved in microRNA biogenesis are unquestionably warranted. Here, we generated Dgcr8 conditional knockout mice by PR-Cre and demonstrated that Dgcr8-dependent canonical microRNAs are critical for uterine morphogenesis and physiological actions of steroid hormones in female reproductive tracts suitable for embryo implantation in mice.

## Results

### Dgcr8 is spatiotemporally deleted in female reproductive tracts of Dgcr8^d/d^ mice in a PR-dependent manner

**Figure 1:**
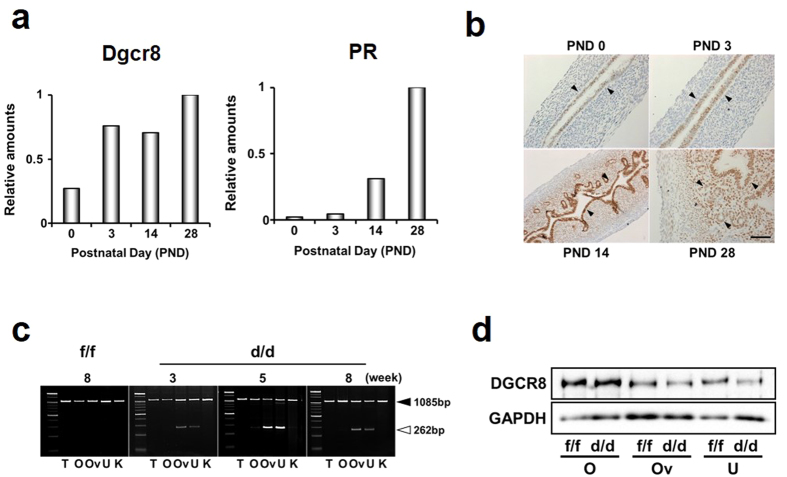
Conditional deletion of Dgcr8 by PR-Cre in female reproductive tracts. (**a**) Temporal expression profiling of Dgcr8 and PR mRNAs in the uterus at various postnatal days (PND) by realtime RT-PCR. Experiments were performed with pooled RNA samples extracted from at least 3 mice for each stage. (**b**) Cell type-specific localization of PR during postnatal uterine development by immunohistochemistry. Arrowheads indicate PR positive cells. (**c**) Representative images of PCR results with genomic DNA of various tissues. Note that Dgcr8 exon 3 is deleted in the uterus and oviduct but not in other tissues examined. Black and white arrowheads indicate PCR products for inclusion (1085 bp) and deletion (262 bp) of Dgcr8 exon 3, respectively. (**d**) Western blotting for DGCR8 protein in the ovaries, oviducts, and uteri of 4-week-old Dgcr8^d/d^ mice. Note that the genomic DNA PCR and Western blotting analyses both show incomplete Dgcr8 deletion in the uterus and oviduct. Scale bar: 50 μm. T, Tail; O, Ovary; Ov, Oviduct; U, Uterus; K; Kidney.

To examine when and where Dgcr8 is deleted in female reproductive tracts, particularly in the uterus of Dgcr8^d/d^ mice, spatiotemporal expression profiles of Dgcr8 and Pgr (PR) in the uterus were first examined (Fig. 1). Realtime RT-PCR results showed that expression levels of Dgcr8 at postnatal day (PND) 3 were already comparable to those at PND 28 while PR expression is very low at PND 0 (birth) and 3 (Fig. 1a,b). The PR protein was merely localized in epithelial cells at PND 3 and its expression became stronger at PND 14. At PND 28, PR was exclusively localized not only in the epithelium, but also in the stroma (Fig. 1b). As the animal sexually matures, PR is not only detected in the epithelial compartments, but also in the sub-epithelial stroma and myometrium^13^. These results were consistent with those of genotyping PCR for Dgcr8^d/d^ mice at various stages (Fig. 1c). While we could barely detect PCR products for deleted allele(s) of Dgcr8 prior to 3 weeks of age, they were prominent from 5 weeks of age. Western blotting supported the inference that uterine deletion of Dgcr8 is PR-dependent (Fig. 1d).

### Dgcr8^d/d^ mice are infertile with acyclic estrous cycles

**Figure 2:**
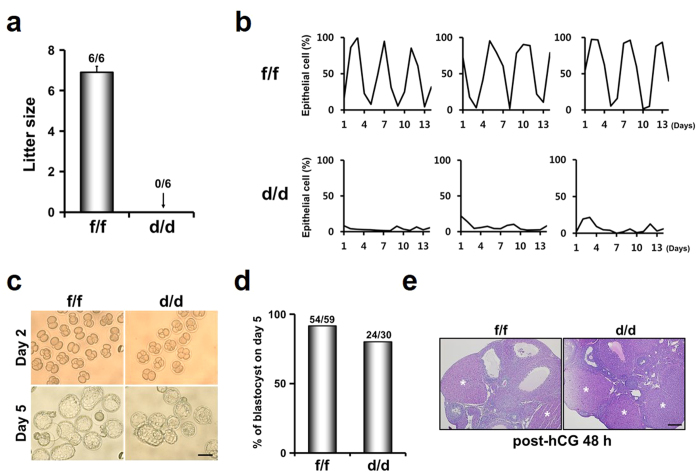
Acyclic infertility of Dgcr8^d/d^ (Dgcr8^f/f^;PR^cre/+^) mice with normal ovarian function. (**a**) Pregnancy outcomes in Dgcr8^f/f^ and Dgcr8^d/d^ mice. Numbers above bars indicate the number of mice with litters/total number of mice examined. (**b**) Examination of estrous cycle in Dgcr8^d/d^ mice. Graphs show % change in epithelial cells/total cells observed on slides by a vaginal smear method for a 2-week period (n = 3 to 8 per each genotype). Note that a cyclic change of the epithelial cell percentage did not occur in Dgcr8^d/d^ mice. (**c,d**) Comparable ovulation, fertilization and embryo development in Dgcr8^d/d^ mice. Pregnancy of 8-week-old Dgcr8^f/f^ and Dgcr8^d/d^ mice (n = 4 per each genotype) was induced by a superovulation regime with PMSG followed by hCG and mating with wild-type mature males with proven fertility. Microscopic images of 2-cell embryos flushed from oviducts at post-hCG 48 h (Day 2) and blastocysts cultured from the 2-cell stage for 3 days *in vitro* (Day 5). The percentage of 2-cell embryos that developed to blastocysts *in vitro* was not different from each other. The numbers above the bars indicate the number of blastocysts/total number of embryo. scale bar: 100 μm. (**e**) Histological analyses for the ovaries of Dgcr8^f/f^ and Dgcr8^d/d^ mice collected at post-hCG 48 h. Note that the corpus luteum (CL) in Dgcr8^d/d^ mice was similar to that of control Dgcr8^f/f^ mice. * indicates CL corpus luteum, scale bar: 200 μm.

To examine whether Dgcr8^d/d^ mice are fertile, they were mated with mature males with proven fertility for 2 months (Fig. 2a). Dgcr8^d/d^ mice did not produce any litters although Dgcr8^f/f^ female mice produced 9 litters (6.9 ± 0.3 pups per litter). By monitoring estrous cycles with daily vaginal smears over a 2-week period, we observed that Dgcr8^d/d^ mice are anestrus whereas Dgcr8^f/f^ mice showed regular estrous cycles with respect to changes in the number of epithelial cells (Fig. 2b).

### Dgcr8^d/d^ mice produce fertilizable oocytes with normal corpus lutea

Previous reports have shown that PR is expressed in the pituitary as well as in female reproductive tracts^13^. Gross histology of the anterior pituitary in Dgcr8^d/d^ mice was not different from that of Dgcr8^f/f^ mice. RT-PCR for FSHβ and LHβ subunits showed production of gonadotropins in the pituitary of Dgcr8^d/d^ female mice (Supplementary Fig. 1). Furthermore, growing follicles at various stages were found in these mice (Data not shown). Thus, to examine whether aberrant control of gonadotropins in the pituitary may cause acyclic sterility in these mice, they were given a superovulation regime with exogenous gonadotropins and mated with fertile males. The total number of 2-cell embryos collected and the percentage of 2-cell embryos that developed to the blastocyst stage during *in vitro* culture were similar in Dgcr8^f/f^ and Dgcr8^d/d^ mice (Fig. 2c,d). In addition, histological analyses of ovaries at post-human chorionic gonadotropin (hCG) 48 h showed that the ovaries of Dgcr8^d/d^ mice had morphologically normal corpora lutea (Fig. 2e). Collectively, these results suggest that the acyclic sterility of Dgcr8^d/d^ mice is not related to intrinsic defects in Dgcr8 deficient ovaries.

### Acute inflammatory infiltration of immune cells occurs in pregnant Dgcr8^d/d^ mice

**Figure 3:**
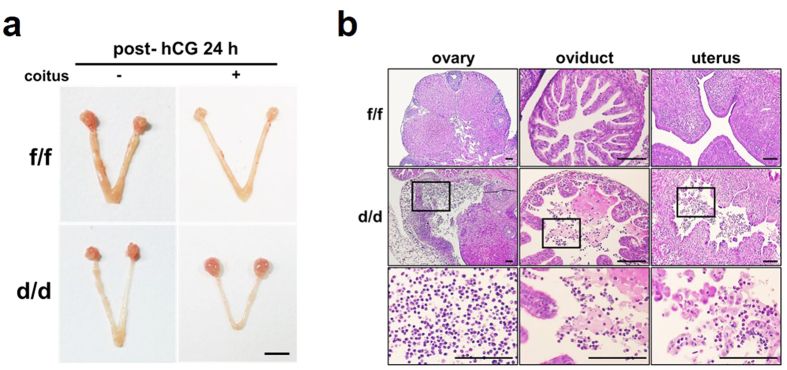
Aberrant acute immune responses in reproductive organs of pregnant Dgcr8^d/d^ mice. (**a**) Gross morphology of female reproductive organs in Dgcr8^f/f^ and Dgcr8^d/d^ mice at post-hCG 24 h after mating with fertile males or no mating. Scale bar: 5 mm. (**b**) Histological analyses of female reproductive organs shown in (**a**). The bottom panel shows higher-magnification images of the boxed area in the middle panel. Note that acute inflammatory cells such as neutrophils and eosinophils are aberrantly abundant in female reproductive organs only in Dgcr8^d/d^ mice mated with fertile males. Scale bar: 100 μm.

In addition to the acyclic infertility of Dgcr8^d/d^ mice, we observed acute inflammatory infiltration of immune cells into the ovaries, oviducts, and uteri of some Dgcr8^d/d^ mice that were given exogenous gonadotropins to induce pregnancy (Fig. 3a). To examine whether microRNA deficiency causes aberrant acute immune responses in these mice, their reproductive organs were histologically examined at post-hCG 24 h following pregnant mare’s serum gonadotropin (PMSG) (Fig. 3b). Interestingly, while acute infiltration of neutrophils and eosinophils was clearly detected in these mice after they were mated with fertile males, gonadotropin treatments alone did not produce any obvious immune reactions. Furthermore, other insults such as oil did not produce any distinct immune reaction in these mice treated with gonadotropins (Supplementary Fig. 2). Thus, these results suggest that deficiency of canonical microRNAs leads to the recruitment of acute inflammatory cells that are reactive to semen containing numerous foreign antigens.

### Expression of microRNA processing factors is regulated by ovarian steroid hormones

To understand whether microRNA processing is influenced in the uterus by ovarian steroid hormones, estrogen (E_2_) and progesterone (P_4_), we examined expression profiles of not only Dgcr8 but also other factors involved in microRNA biogenesis in uteri of ovariectomized mice exposed to E_2_ and/or P_4_ at various time points. Although the expression of Dgcr8 mRNA is not significantly altered by these hormones, mRNA expression of Dicer, Exportin 5 (Xpo5) and Argonaute 2 (Argo2) among factors examined in this study was temporally regulated by E_2_ (Supplementary Fig. 3). While P_4_ itself did not show any critical actions on expression profiles of these factors, it interfered with estrogen actions on expression patterns of these factors. Furthermore, pretreatment of ICI 182,780, a nuclear estrogen receptor antagonist, inhibited estrogenic actions on expression of these factors (Supplementary Fig. 4). These results collectively suggest that ovarian steroid hormones are involved in regulation of expression of microRNA processing factors with temporal manners in the uterus.

### Onset of multiple uterine abnormalities in Dgcr8^d/d^ mice coincides with cell- and time-specific expression of PR in the uterus

**Figure 4:**
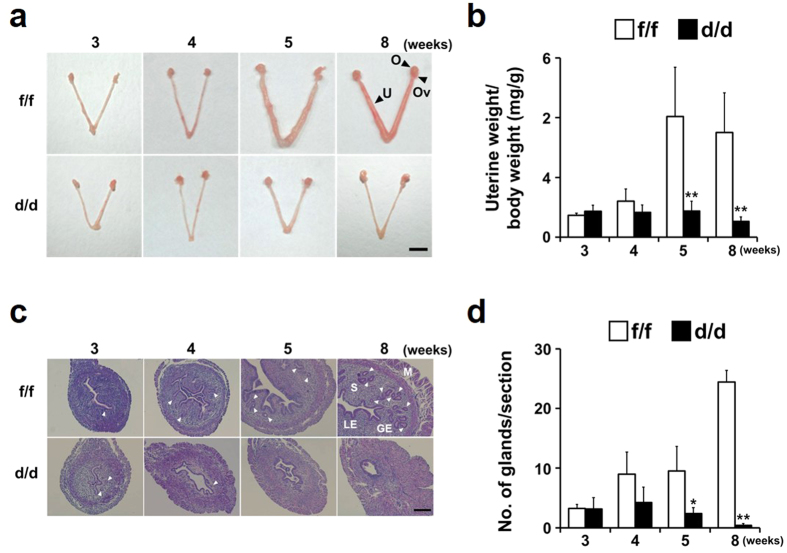
Gross and histological analyses for uterine development of Dgcr8^d/d^ mice at various ages. (**a**) Gross morphology of the uteri of 3- to 8-week-old Dgcr8^d/d^ mice. Note that while the uteri of 3-week-old Dgcr8^d/d^ mice look normal, they become severely abnormal beginning at 5 weeks of age. Scale bar: 5 mm. (**b**) Changes of uterine weight/total body weight of Dgcr8^d/d^ mice during uterine development. Consistent with the distinct morphologic abnormalities observed in mice beginning at 5 weeks of age, uterine weight was dramatically lower than that of control from 5-week-old stage (n = 4 to 6 for each group). (**c**) Representative microscopic images of the uteri of 3- to 8-week-old Dgcr8^f/f^ and Dgcr8^d/d^ mice. Scale bar: 100 μm. (**d**) The number of uterine glands/section observed in Dgcr8^f/f^ and Dgcr8^d/d^ mice (n = 4 to 6 for each group). Unpaired Student’s *t*-test, *p < 0.05, **p < 0.01. O, Ovary; Ov, Oviduct; U, Uterus; M, Myometrium; S, Stroma; GE, Gland epithelium; LE, Luminal epithelium. White arrowheads indicate glands.

To examine whether Dgcr8 deficiency affects uterine development, the uteri of Dgcr8^d/d^ mice were grossly examined at various stages (Fig. 4a). At 3 weeks of age, the gross morphology, histology and uterine weight of Dgcr8^d/d^ mice were similar to those of Dgcr8^f/f^ mice. However, at 4 weeks of age when PR expression is significantly increased both in epithelial and stromal cell compartments (Fig. 1), some Dgcr8^d/d^ mice started to show multiple uterine defects, and the severity of these deformities increased over time. A comparison of uterine weight to body weight at the pre-pubertal and adult stages showed a progressive decrease in the uterine size of Dgcr8^d/d^ mice (Fig. 4b). To further investigate uterine abnormalities in Dgcr8^d/d^ mice, uterine sections of 3- to 8-week-old Dgcr8^d/d^ mice were examined histologically. Consistent with gross morphology, the uteri became severely abnormal at 5 weeks of age whereas it looked normal at 3 weeks of age (Fig. 4c). Furthermore, the number of glands was significantly reduced in the uteri of Dgcr8^d/d^ mice beginning at 5 weeks of age (Fig. 4c,d).

**Figure 5:**
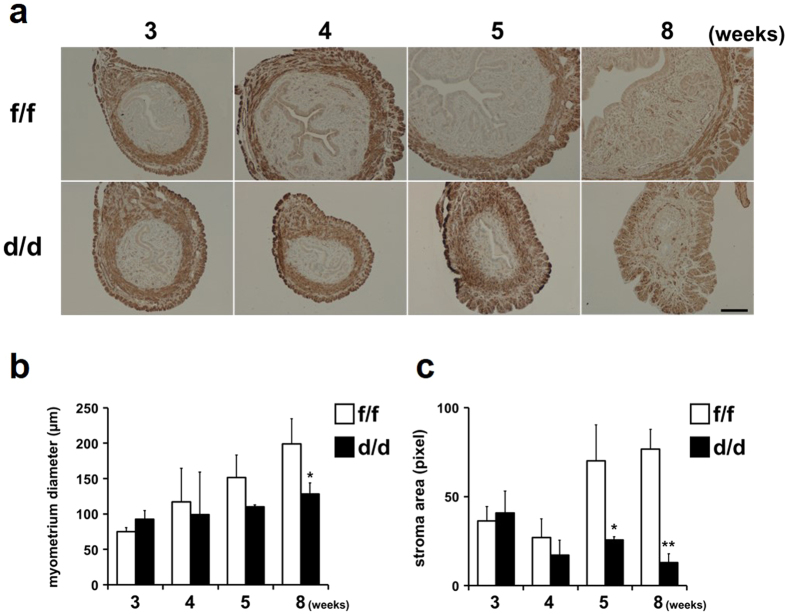
Stromal and myometrial abnormalities during uterine development of Dgcr8^d/d^ mice. (**a**) Immunohistochemical analyses for alpha smooth muscle actin (α-SMA) in the uterine myometrial layers of Dgcr8^d/d^ mice. Dark brown color indicates α-SMA positive smooth muscle cells in the uterus. Scale bar: 100 μm. (**b**) Myometrial thickness in the uteri of Dgcr8^f/f^ and Dgcr8^d/d^ mice (n = 4 to 6 for each group). Myometrial thickness was determined by the length of the area of α-SMA positive cell layers. (**c**) Quantification of stromal area during uterine development in Dgcr8^d/d^ mice. Stromal area was quantitatively measured by pixels of images for each uterine section of Dgcr8^f/f^ and Dgcr8^d/d^ mice. At least three independent sections were microscopically examined for each mouse (n = 4 to 6 mice for each group). Unpaired Student’s *t*-test, *p < 0.05, **p < 0.01.

An immunohistochemical analysis for alpha smooth muscle actin (α-SMA), a smooth muscle cell-specific marker, was performed to examine whether a loss of Dgcr8 causes any abnormalities in the uterine myometrium (Fig. 5a,b). The myometrial thickness evaluated by α-SMA was more or less similar to that of Dgcr8^f/f^ mice at pre-pubertal stages, as expected from the PR expression patterns. However, it became significantly reduced at 8-week-old Dgcr8^d/d^ mice since the inner circular smooth muscle layer suddenly became narrow. Furthermore, the stromal cell compartment gradually dwindled in Dgcr8^d/d^ mice throughout uterine development whereas it became noticeably expanded in Dgcr8^f/f^ mice between 4 and 5 weeks of age (Fig. 5c). Collectively, these results suggest that the development of all three major uterine cell compartments is severely impaired in Dgcr8^d/d^ mice.

### Disturbed hormone responses in stromal cells cause aberrantly reduced cell proliferation in Dgcr8^d/d^ mice

**Figure 6:**
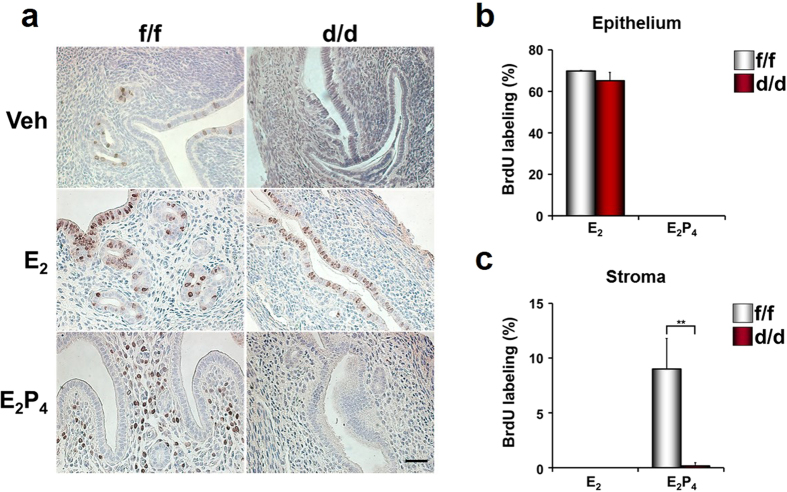
Cell proliferation in the uteri of ovariectomized Dgcr8^d/d^ mice treated with ovarian steroid hormones. (**a**) BrdU incorporation experiments were performed to examine hormone-dependent endometrial cell proliferation in Dgcr8^d/d^ mice. Ovariectomized mice were sacrificed 24 h after injection with vehicle, E_2_ or E_2_ + P_4_. BrdU was given to these mice 3 h before sacrifice. Brown color indicates the nuclei of BrdU-incorporated cells. Scale bar: 25 μm. (**b,c**) Graphs depicting the percentage of BrdU positive cells/total number of cells counted. Note that stromal cell proliferation was severely impaired in Dgcr8^d/d^ mice, whereas uterine epithelial cells normally responded to E_2_. Unpaired Student’s *t*-test, *p < 0.05, **p < 0.01.

**Figure 7:**
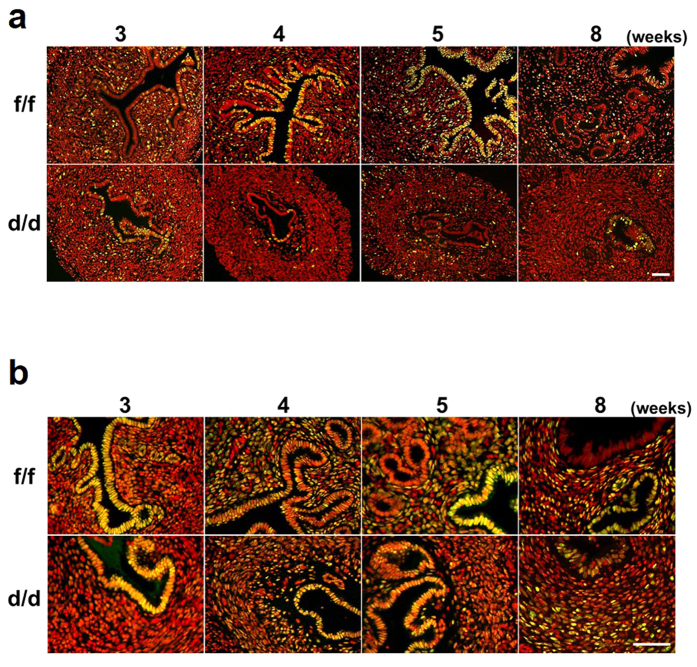
Expression of Ki-67 and PR in uteri of Dgcr8^d/d^ mice at various stages. (**a,b**) Immunofluorescence of Ki-67 (**a**) and PR (**b**) on uterine sections from Dgcr8^f/f^ and Dgcr8^d/d^ mice at various ages. Ki-67 or PR was visualized as green and nuclei were stained with TO-PRO-3-Iodide (red). Yellow color indicates Ki-67 or PR positive cells. Scale bar: 50 μm.

**Figure 8:**
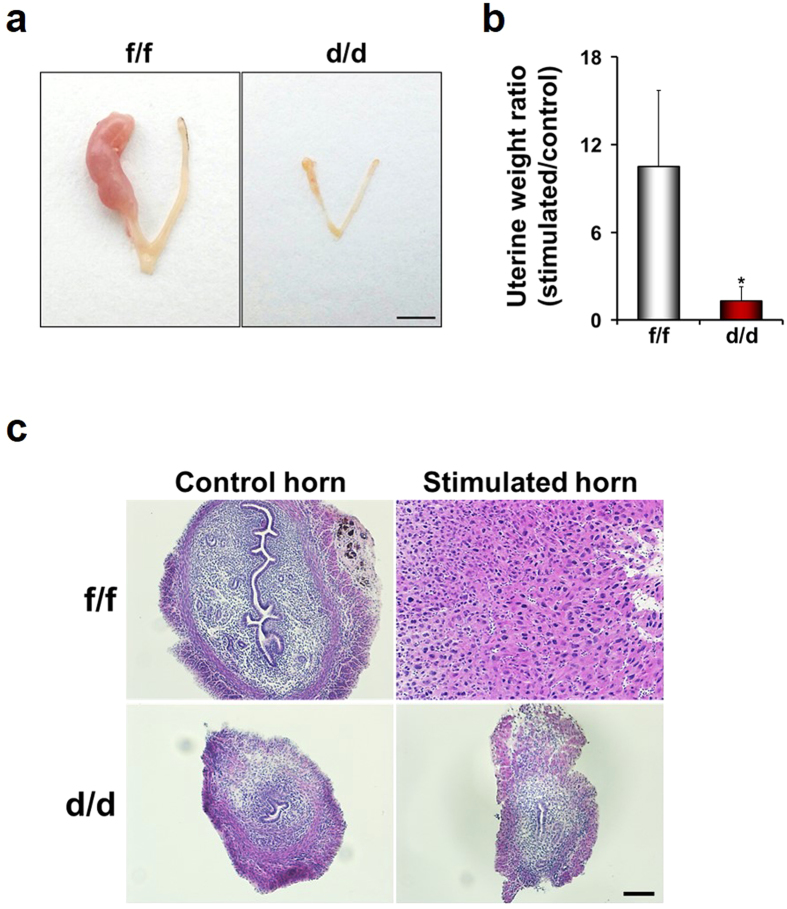
Impaired decidualization of Dgcr8^d/d^ mice. (**a**) Gross anatomy of Dgcr8^d/d^ uterus 4 days after decidualization was artificially induced by injecting oil into a uterine horn. Scale bar: 5 mm. (**b**) Decidual response was determined by uterine weight of stimulated horn/control horn. (n = 4 to 5 for each group). Unpaired Student's *t*-test, *p < 0.05. (**c**) Representative microscopic images of the uteri of Dgcr8^f/f^ and Dgcr8^d/d^ mice traumatized with oil. Scale bar: 100 μm.

To examine whether multiple uterine abnormalities could be due to aberrant response(s) to ovarian steroid hormones, E_2_ and P_4_, BrdU incorporation experiments were performed with ovariectomized mice treated with vehicle, E_2_ or E_2_ + P_4_ (Fig. 6)^14^,^15^. E_2_ induced epithelial cell proliferation in both Dgcr8^f/f^ and Dgcr8^d/d^ mice. However, stromal cells did not undergo proliferation at all in Dgcr8^d/d^ mice under the influence of E_2_ + P_4_, whereas Dgcr8^f/f^ mice showed typical stromal cell proliferation. Furthermore, Ki-67 immunofluorescence staining indicated that stromal cell proliferation is impaired in these mice throughout uterine development (Fig. 7a). Consistent with the impaired stromal cell proliferation (Figs 6 and 7), uterine stromal cells of Dgcr8^d/d^ mice pretreated with ovarian steroid hormones failed to show signs of decidualization when it was experimentally-induced by giving an oil injection into one of the uterine horns (Fig. 8).

Since aberrantly reduced PR expression could be a major cause of abnormal P_4_ responses in Dicer^flox/flox^;PR^cre/+^ mice^12^, we examined whether PR is appropriately expressed in these mice during uterine development (Fig. 7b). In contrast to the significant reduction of uterine PR expression in Dicer^flox/flox^;PR^cre/+^ mice^12^, PR was clearly detectable in all three major uterine cell types in Dgcr8^d/d^ mice, similar to its expression in Dgcr8^f/f^ mice during uterine development. Collectively, these results suggest that Dgcr8 deficient stromal cells are unable to properly respond to ovarian steroid hormones, leading to severe stromal atrophy. To investigate whether severe atrophy in stromal cells of Dgcr8^d/d^ mice is affected by facilitated apoptosis, TUNEL assays were performed in 3- and 5-week-old mice. Few TUNEL positive cells were detected in both Dgcr8^f/f^ and Dgcr8^d/d^ mice (Supplementary Fig. 5). Furthermore, the expression levels of apoptosis-related genes such as Bcl2l11, Aldh1a3 and Fas were not significantly different between them. These results suggest that severe atrophy in the stromal cell compartment of Dgcr8^d/d^ uteri is not associated with facilitated apoptosis.

## Discussion

The endometrium is a complex tissue that undergoes cyclic proliferation and differentiation under the influence of ovarian steroid hormones, E_2_ and P_4_. Imbalance between the actions and levels of the major regulators of endometrial function may cause several pathophysiological conditions such as endometriosis and endometrial cancer^16^,^17^. MicroRNAs have been proposed to regulate the stability of mRNAs that encode proteins involved in maintaining normal physiological processes such as cell proliferation and differentiation, apoptosis, angiogenesis, and inflammation^18^,^19^. Recent studies have demonstrated that dysregulated microRNA expression as well as hormonal discrepancy is also important for incidence of these conditions in the uterus^20^,^21^.

Previous reports have suggested that ovarian steroid hormones regulate expression profiles of factors participating in microRNA biogenesis as wells as many microRNAs in the uterus^22^,^23^,^24^. It was shown that E_2_ and P_4_ significantly increase the levels of Xpo5 mRNA, but only P_4_ increases Dicer expression^22^. However, E_2_ but not P_4_ influenced the expression patterns of Xpo5 and Dicer at the mRNA level in this study (Supplementary Fig. 3). In addition, mRNA expression of Argo2, a member of the Argonaute family, showed similar patterns to those of Dicer and Xpo5 after E_2_ treatment. While P_4_ did not have significant action on expression of all the genes examined in the uterus, it seemed to dampen and/or delay the action of E_2_ on the expression of Dicer, Xpo5, and Argo2 (Supplementary Fig. 3). Although there are several incompatible results between a previous work^22^ and this study, both demonstrated that action(s) of steroid hormones on the expression patterns of these genes are mediated via their nuclear receptors (Supplementary Fig. 4). To further understand exact actions of steroid hormones on the expression profiles of factors involved in microRNA biogenesis, cell type-specific localization of these factors in the uterus under the influence of ovarian steroid hormones needs to be examined.

Two Cre transgenic lines, Amhr2^cre/+^ (Amhr2-Cre) and PR^cre/+^ (PR-Cre), have been mainly utilized to delete genes of interest in female reproductive tracts^9^,^10^,^11^,^12^. Amhr2-Cre is expressed embryonically in the mesenchyme of the developing Mullerian ducts, and postnatally in ovarian granulosa cells and the stromal and myometrial layers of female reproductive tracts^25^,^26^,^27^. As expected from Amhr2 expression profiles, Dicer^flox/flox^;Amhr2^cre/+^ mice are infertile due to multiple defects in female reproductive tracts such as disorganized oviducts with cysts and shorter uterine horns, although these mice have normal mating behavior^9^,^10^,^11^ that is not shown in Dgcr8^d/d^ mice (Fig. 2).

**Figure 9:**
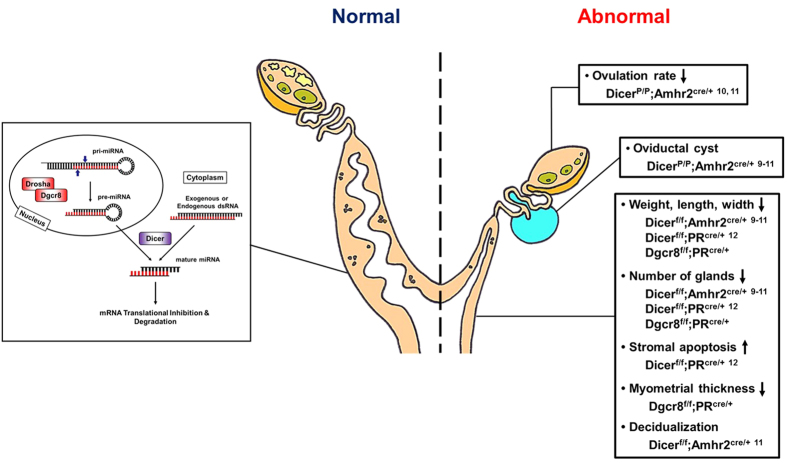
A schematic cartoon to summarize major reproductive phenotypes of Dicer or Dgcr8 conditional knockout mice by Amhr2-Cre or PR-Cre.

With respect to the severity and types of phenotypes in the uterus, conditional deletion of Dicer or Dgcr8 by PR-Cre led to more severe abnormalities than those in Dicer^flox/flox^;Amhr2^cre/+^ mice. For example, decidualization was normally induced in Dicer^flox/flox^;Amhr2^cre/+^ mice^11^, but completely failed in Dgcr8^d/d^ mice (Fig. 8). In addition, the inner circular smooth muscle layer seems to be suddenly disintegrated in adult Dgcr8^d/d^ mice (Fig. 5). These differences could reflect the usage of different CRE systems in the uterus, considering that Dgcr8^d/d^ mice show various uterine defects that were shared with Dicer^flox/flox^;PR^cre/+^ mice. The reproductive abnormalities have many similarities, including shorter uterine horns, a reduced number of glands, and severe stromal atrophy with aberrant hormone responsiveness (Fig. 9), although the onset of reproductive phenotypes is somewhat different between Dicer^flox/flox^;PR^cre/+^ mice^12^ and Dgcr8^d/d^ mice. These results indicate that canonical microRNAs are critical for postnatal uterine development, architecture, and physiological function. Moreover, Dicer^flox/flox^;PR^cre/+^ mice^12^ and Dgcr8^d/d^ mice share stromal atrophy with aberrant progesterone signaling (Fig. 6), a unique phenotype that did not occur in Dicer^flox/flox^;Amhr2^cre/+^ mice.

A growing number of publications have provided evidence that specific microRNAs in many species, including humans and mice, are essential for the sequential events of embryo implantation from embryo-epithelial juxtacrine adhesion to decidualization by regulating critical factors for embryo implantation such as IGF1R, MUC1, LIF and COX-2^28^,^29^,^30^,^31^,^32^. While DGCR8 expression is not altered in the uterus during early pregnancy, DROSHA expression is increased during the decidualization of stromal cells *in vitro*^24^, implicating the importance of microRNAs during these events. In this respect, it is interesting that knockdown of Dicer dysregulates expression of only a few dozens of microRNAs and has only a minor effect on human endometrial stromal cells during *in vitro* decidualization^33^. Furthermore, Dicer deficiency causes a minor alteration in the microRNA signature in the oviducts (Dicer^flox/flox^;Amhr2^cre/+^) and uteri (Dicer^flox/flox^;PR^cre/+^) of Dicer conditional knockout mice^9^,^10^,^11^,^12^, suggesting that a large cohort of microRNAs could be produced in female reproductive tracts by non-canonical microRNA processing.

Unexpectedly, we observed acute immune cell infiltration in Dgcr8^d/d^ mice mated with males for induction of pregnancy after a superovulation regime was given (Fig. 3). Administration of exogenous gonadotropins itself did not provoke any acute inflammatory responses unless Dgcr8^d/d^ female mice were mated with fertile males. This suggests that microRNAs, especially those regulated by Microprocessor complex, could be a participant for immune modulation in these organs. MicroRNA-dependent signaling pathways may modulate cytokines to provoke immune cell infiltration, which can be caused by semen containing various foreign antigens. In addition, immune cells deficient of microRNAs may act aberrantly for themselves since immune cells such as T cells also express PR^34^,^35^. This phenotype is somewhat different from the chronic inflammation observed in immature non-pregnant Dicer^flox/flox^;Amhr2^cre/+^ mice^9^. Further studies are warranted to delineate the regulatory action(s) of microRNAs on the aberrant immune responses in these organs.

Since DICER is involved in the production of not only canonical microRNAs but also endo siRNAs, non-canonical microRNAs, and other small RNAs in mammals^4^,^36^, Dgcr8^d/d^ mice could be an alternative animal model to understand the precise action(s) of microRNAs in mammals. In fact, to clarify the distinct physiological roles of microRNAs and endo siRNAs, several studies have compared the phenotypes of Dicer and Dgcr8 conditional knockout mice with tissue- or cell type-specific manners^37^,^38^,^39^,^40^. Our results in Dgcr8^d/d^ mice reinforced the notion that reproductive abnormalities in the oviducts and uteri of Dicer conditional knockout mice with either PR-Cre or Amhr2-Cre are related to deficiencies in canonical microRNAs. However, several unique phenotypes including myometrial defect were observed in Dgcr8^d/d^ mice (Fig. 5b). Moreover, Dgcr8^flox/flox^;Amhr2^cre/+^ mice show subfertility with smaller litter size which has not been observed in any Dicer or Dgcr8 conditional knockout mice to date (Kim *et al*. in preparation). These data suggest a possibility that the Microprocessor complex containing DGCR8 may have a distinct action other than canonical microRNA processing. In fact, there was an interesting report that the Microprocessor complex negatively regulates long interspersed element-1 and Alu retrotransposons in microRNA- and DICER-independent manners^41^. Furthermore, a recent study has provided evidence that DGCR8 itself acts for neural morphogenesis in a DROSHA-independent way in *Drosophila*^42^. Additional studies are definitely requested to determine whether DGCR8 has unique functions that are not associated with the sequential events of microRNA processing. In summary, here we provide a valuable animal model deficient of Dgcr8 that have similar and distinct reproductive phenotypes compared with Dicer deficient mice. Our results clearly demonstrate that canonical microRNAs are critical for uterine morphogenesis and the physiological actions of steroid hormones in female reproductive tracts suitable for embryo implantation in mice.

## Methods

### Animals and genotyping of Dgcr8^d/d^ mice

All animal experiments were carried out in accordance with the protocols approved by the Institutional Animal Care and Use Committee of CHA University (IACUC, No 140005). All animals used in this study were maintained and handled according to the policies approved by CHA University. Six to 8-week-old adult ICR mice were provided by Orient Bio (Gapyeong, Gyeonggi, Korea).

Dgcr8^flox/flox^ mice were initially generated and provided by Dr. Elaine Fuchs’ laboratory^43^. Genotyping PCR was performed using genomic DNA extracts from mouse tail biopsies. Dgcr8^flox/flox^ mice were bred to PR^cre/+^ (herein called PR-Cre) mice^13^ to generate Dgcr8^flox/+^ ;PR^cre/+^ mice. These mice were then crossed to generate Dgcr8^flox/flox^;PR^cre/+^ (designated as Dgcr8^d/d^ mice hereafter) and Dgcr8^flox/flox^;PR^+ /+^mice (Dgcr8^f/f^ mice). To distinguish the Dgcr8 null allele (Dgcr8^d/d^) from Dgcr8^f/f^, primers were designed to amplify 262 bp (deletion of exon 3) and 1085 bp (presence of exon 3) PCR products as shown in Fig. 1c.

### RNA extraction, reverse transcription-PCR (RT-PCR) and realtime RT-PCR

Total RNA was extracted from mouse uterus by using Trizol Reagent (Invitrogen Life Technologies, San Diego, CA, USA) according to the manufacturer’s protocols. The first-strand cDNA was synthesized from 2 μg of total RNA by using M-MLV reverse transcriptase (Promega, Madison, WI, USA) and RNasin Ribonuclease Inhibitor (Promega). The synthesized cDNA was utilized for PCR with specific primers at optimized cycles. For quantification of expression level, realtime RT-PCR was performed by using iQ™ SYBR Green Supermix (Bio-Rad, Hercules, CA, USA) on a BIO-RAD iCycler. To compare transcript levels between samples, a standard curve of cycle thresholds for several serial dilutions of a cDNA sample was established and then used to calculate the relative abundance of each gene. rPL7 was used as a reference gene in all the experiments performed in this study. All PCR reactions were performed in duplicate.

### Western blotting

Tissues were lysed in lysis buffer including PRO-PREP (iNtRON, Seongnam, Korea) solution and 1X phosphatase inhibitor (Roche Applied Sciences, Indianapolis, IN, USA). The protein samples (10 μg/lane) were then separated by 8% SDS-PAGE, transferred onto nitrocellulose membrane (Bio-Rad) and blocked with 5% non-fat milk (Bio-Rad) in TBS (Bio-Rad) containing 0.1% Tween 20 (Sigma-Aldrich, St. Louis, MO, USA). Anti-DGCR8 (Proteintech, Chicago, IL, USA, 1:100) and anti-GAPDH (Cell Signaling, Danvers, MA, USA, 1:2000) were used for Western blotting analyses. The signals were developed using an ECL Western blotting substrate kit (Bio-Rad) and detected using a Chemidoc XRS + (Bio-Rad) with Image Lab software.

### Vaginal smear assay and fertility analysis

Vaginal smears from 6- to 7-week-old Dgcr8^f/f^ and Dgcr8^d/d^ mice were collected daily over a 2-week period as previously described^44^. The stage of the estrous cycle (proestrus, estrus, metestrus, and diestrus) was determined based on the presence or absence of leukocytes, cornified epithelial cells, and nucleated epithelial cells. To evaluate reproductive performance, 8-week-old Dgcr8^f/f^ and Dgcr8^d/d^ mice (n = 6 for each genotype) were individually bred to males with proven fertility. The numbers of litters and pups were recorded for a 2-month period.

### Superovulation and preimplantation embryo culture

To induce ovulation, 8-week-old mice were given intraperitoneal (i.p) injections of 5 IU PMSG (Sigma-Aldrich) for 48 h, followed by i.p injections with 5 IU hCG (Sigma-Aldrich). Mice were then bred to wild-type males with known fertility and pregnancy was evaluated by the presence of vaginal plug next morning. The 2-cell embryos flushed from oviducts at post-hCG 48 h were cultured up to the blastocyst stage in 20 μl droplets of KSOM (Millipore, Danvers, MA, USA) covered with oil (SAGE *In-Vitro* Fertilization, Inc., Trumbull, CT, USA) in a petri dish.

### Tissue collection, H&E staining and immunostaining

Female reproductive organs were dissected, and then fixed in 4% paraformaldehyde for histology or snap frozen for RNA and/or protein preparation. Fixed tissues were washed, dehydrated, and embedded in Paraplast (Merck KGaA, Darmstadt, Germany). Paraffin-embedded tissues were sectioned using a microtome, stained with hematoxylin and eosin (H&E) (Sigma-Aldrich), and observed by a light microscopy.

For immunostaining analyses, antibodies specific to PR (Thermo Scientific, Pierce Biotechnology, Rockford, IL, USA, 1:100), α-SMA (Abcam, Cambridge, UK, 1:100), and Ki-67 (Abcam, 1:100) were used in 5 μm thick paraffin-embedded sections. Blocking was carried out using protein block serum (Dako, Carpinteria, CA, USA) for 1 h, and then samples were incubated with an appropriate primary antibody at 4 °C overnight. On the following day, sections were washed in PBS and incubated with HRP secondary antibody or FITC conjugated donkey anti-rabbit IgG (Jackson ImmunoResearch, West Grove, PA, USA) for 1 h at room temperature. Nuclear staining was performed using TO-PRO-3-iodide (Life Technologies, Carlsbad, CA, USA). For Immunohistochemistry, DAB reagent (Vector Laboratories, Inc., Burlingame, CA, USA) was applied to visualize signals. Slides were counterstained with hematoxylin, mounted with mounting solution and cover-slipped.

### Quantitative measurement of the number of uterine glands and the thickness of the uterine smooth muscle layer in Dgcr8^d/d^ mice

To quantitatively examine uterine gland formation in Dgcr8^d/d^ mice, 4 independent uterine cross-sections for each 3- to 8-week-old mouse were H&E stained and the number of uterine glands was counted in a blind manner (n = 3 to 5 mice per genotype in each age group). Immunohistochemical staining for α-SMA, as a smooth muscle cell marker, was performed to evaluate the thickness of myometrial layers of Dgcr8^d/d^ uteri. The captured microscopic images of α-SMA staining were applied to Image J software (National Institutes of Health, Bethesda, MD, USA) for measuring the thickness of myometrial layers of Dgcr8^d/d^ mice at each age.

### BrdU incorporation and TUNEL assays

Eight-week-old Dgcr8^f/f^ and Dgcr8^d/d^ mice were ovariectomized, rested for 2 weeks, and treated subcutaneously with either 0.1 ml sesame oil as vehicle (Acros, NJ, USA), 200 ng E_2_ (Sigma-Aldrich), or 200 ng E_2_ + 2 mg P_4_ (Sigma-Aldrich). Mice were sacrificed 24 h after steroid hormone treatment(s). 5-Bromo-2´-Deoxyuridine (BrdU) (Invitrogen Life Technologies) was given to the mice 3 h before they were sacrificed. Uteri were dissected, fixed in 4% paraformaldehyde and then subjected to paraffin-embedded tissue processing. Uterine sections were utilized for BrdU immunostaining using a BrdU staining kit (Invitrogen Life Technologies), according to the manufacturer’s instructions.

Uterine apoptosis of Dgcr8^d/d^ mice was assessed using an *In Situ* Cell Death Detection Kit according to the manufacturer instructions (Roche, West Sussex, UK). Sections were deparaffinized and rehydrated in a graded alcohol series, and then processed for antigen retrieval. They were incubated with TUNEL reaction mixture for 1 h at 37 °C and then with DAPI for 10 min at room temperature, and observed under a fluorescence microscope.

### Artificial decidualization

Briefly, 8-week-old female mice were ovariectomized and rested for 2 weeks, and then received daily injections of 100 ng E_2_ for 3 days. After 2 days of resting, mice were then treated with daily injections of 1 mg P_4_ and 6.7 ng E_2_ for 3 days. At 6 h after the last injection, one uterine horn was traumatized by the injection of 50 μl of sesame oil. Mice were given daily injections of 1 mg P_4_ and 6.7 ng E_2_/mouse following trauma. Mice were sacrificed at day 4 after the trauma.

### Statistics

Realtime RT-PCR was performed in duplicate for at least three independent samples. All values represent the mean ± standard deviation. Unpaired Student’s *t*-tests were used for statistical evaluation and p < 0.05 was considered statistically significant.

## Additional Information

**How to cite this article**: Kim, Y. S. *et al*. Deficiency in DGCR8-dependent canonical microRNAs causes infertility due to multiple abnormalities during uterine development in mice. *Sci. Rep.* **6**, 20242; doi: 10.1038/srep20242 (2016).

## Supplementary Material

Supplementary Information
