# Supplementary material for: Deficiency in DGCR8-dependent canonical microRNAs causes infertility due to multiple abnormalities during uterine development in mice
Source: Sci Rep. 2016 Feb 2;6:20242. doi: 10.1038/srep20242 (PMC4735737; doi:10.1038/srep20242)
Supplement: Supplementary Information [file srep20242-s1.pdf]

# Deficiency in DGCR8-dependent canonical microRNAs causes infertility due to multiple abnormalities during uterine development in mice

Yeon Sun Kim[1], Hye-Ryun Kim[1], Hyongbum Kim[2], Seung Chel Yang[1], Mira Park[1], Jung Ah Yoon[3], Hyunjung J Lim[4], Seok-Ho Hong[5], Francesco J DeMayo[6], John P Lydon[6], Youngsok Choi[1], Dong Ryul Lee[1,3], Haengseok Song[1,3,*]

[1]Department of Biomedical Science, CHA University, Seongnam, Gyeonggi, Korea

[2]Department of Pharmacology and Brain Korea 21 PLUS Project for Medical Science, Yonsei University College of Medicine, Seoul 120-752, Korea

[3]Fertility Center of CHA Gangnam Medical Center, CHA University, Seoul, Korea

[4]Department of Veterinary Medicine, College of Veterinary Medicine, Konkuk University, Seoul, 143-701 Korea

[5]Department of Internal Medicine, School of Medicine, Stem Cell Institute, Kangwon National University, Chuncheon, Kangwon 200-701, Korea

[6]Department of Molecular and Cellular Biology, Baylor College of Medicine, Houston, Texas, USA

*Correspondence should be addressed to: H Song, Ph.D. (hssong@cha.ac.kr) at Department of Biomedical Science, CHA University (Tel: 82-31-881-7150, Fax: 82-31-881-7249).

**Figure S1. Gross histology and gonadotropin production in Dgcr8$^{d/d}$ mice.** Gross histology (a) and RT-PCR results for FSHβ and LHβ subunits (b) expressed in the pituitary of Dgcr8$^{d/d}$ mice. The bottom panel in (a) shows higher-magnification images of the boxed area in the top panel. Scale bar: 50 μm.

**Figure S2. Histologic examination for acute immune responses in reproductive organs of Dgcr8$^{d/d}$ mice injected with oil.** (a) Gross morphology of female reproductive organs in Dgcr8$^{f/f}$ and Dgcr8$^{d/d}$ mice 12 h after oil injection into uterine horns at post-hCG 9 h. Scale bar: 5 mm. (b) Histological analyses of female reproductive organs shown in (a). Note that there are no inflammatory cells such as neutrophils and eosinophils presented in Figure 3. Scale bar: 100 μm.

**Figure S3. Expression profiles of miRNA biogenesis factors in mouse uterus.** RT-PCR (a) and realtime RT-PCR (b) analyses for relative mRNA levels of miRNA biogenesis factors by $E_2$ (200 ng) and/or $P_4$ (2 mg) in the uteri of ovariectomized mice at different time points (n=5 for each group). Egr1 and Ltf were used as positive controls for early and late estrogen responses, respectively. Areg and Hoxa10, and Ptgs1 were for $P_4$ and $E_2$+$P_4$ responses, respectively.

**Figure S4. Analyses of $E_2$-dependent expression of miRNA biogenesis components via nuclear estrogen receptor(s).** Expression patterns of Dicer, Argo2 and Xpo5 were analyzed by RT-PCR (a) and realtime RT-PCR (b-d) to examine whether nuclear estrogen receptor(s) are involved in $E_2$-dependent expression of these genes. Ovariectomized mice were treated with ICI 182, 780, a nuclear estrogen receptor antagonist, 30 min before $E_2$ (200 ng) treatments and sacrificed 3 h after $E_2$ administration (n=4 to 7 for each group). Unpaired Student *t*-test, *=p<0.05.

**Figure S5. TUNEL assays for the uterine sections of 3- and 5-week-old Dgcr8$^{d/d}$ mice.** (a) Microscopic images for apoptotic cells (green) counterstained with DAPI (blue) in uterine sections from Dgcr8$^{f/f}$ and Dgcr8$^{d/d}$ mice. Note that uteri of both Dgcr8$^{f/f}$ and Dgcr8$^{d/d}$ mice at 3 and 5 weeks of age were negative in the TUNEL assays. Scale bar: 50 μm. (b) Realtime RT-PCR for expression of apoptosis marker genes (Bcl2l11, Aldh1a3, and Fas) in the uteri of 3- and 5-week-old Dgcr8$^{f/f}$ and Dgcr8$^{d/d}$ mice.

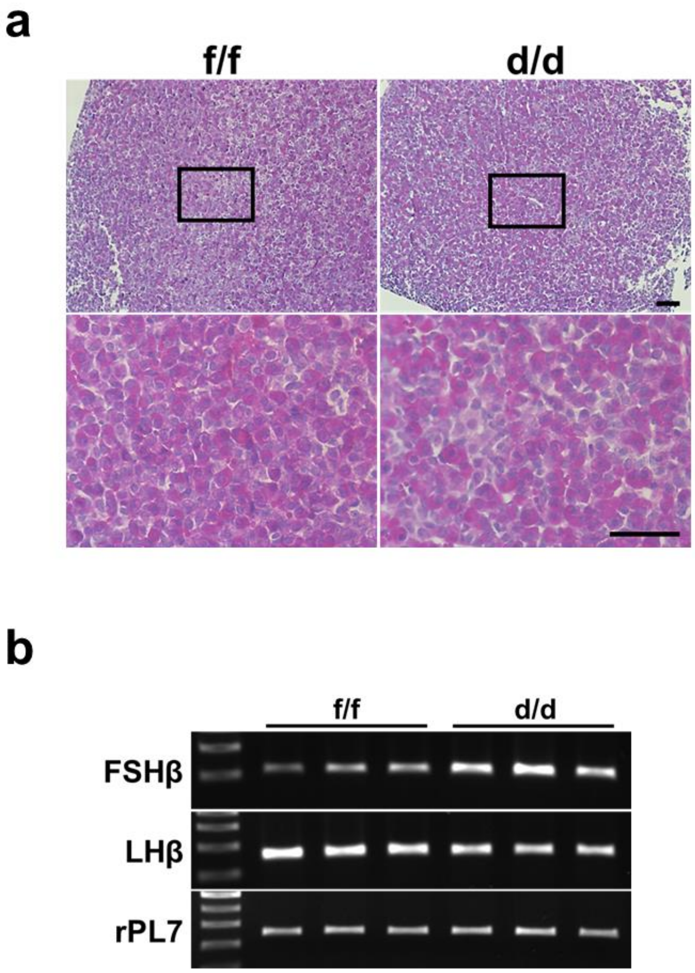
a
f/f
d/d
b
f/f
d/d
FSHβ
LHβ
rPL7

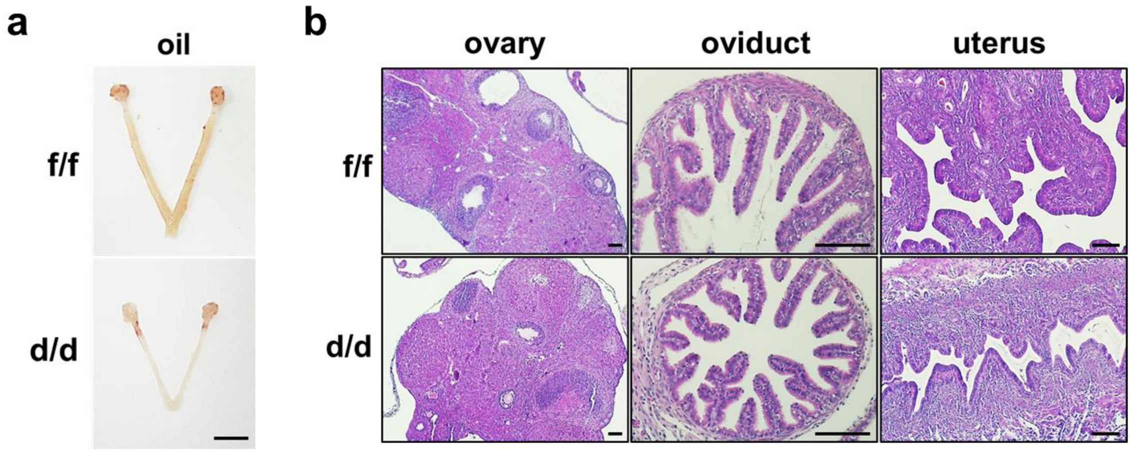
a
oil
f/f
d/d
b
ovary
oviduct
uterus
f/f
d/d

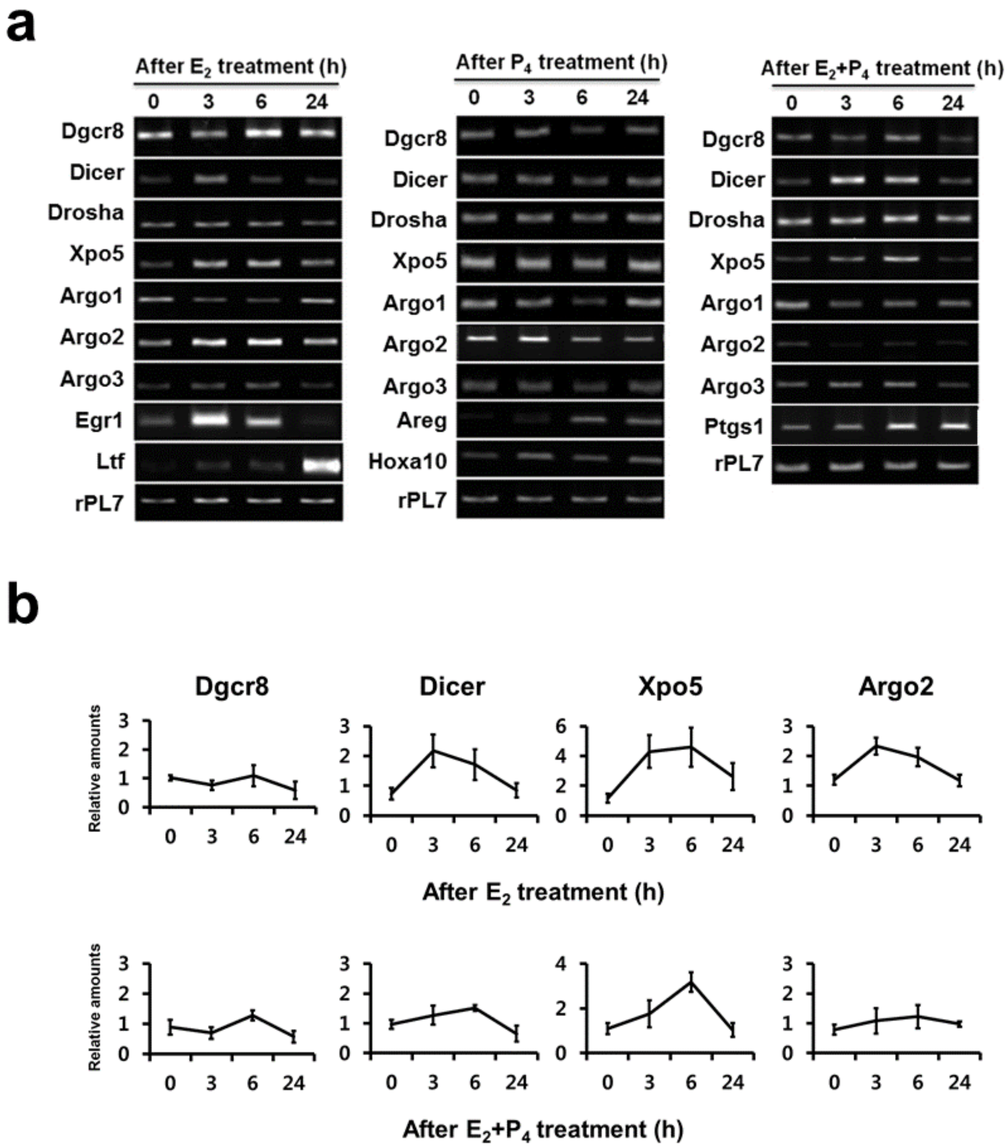
a
After E2 treatment (h)
0 3 6 24
Dgcr8
Dicer
Drosha
Xpo5
Argo1
Argo2
Argo3
Egr1
Ltf
rPL7
After P4 treatment (h)
0 3 6 24
Dgcr8
Dicer
Drosha
Xpo5
Argo1
Argo2
Argo3
Areg
Hoxa10
rPL7
After E2+P4 treatment (h)
0 3 6 24
Dgcr8
Dicer
Drosha
Xpo5
Argo1
Argo2
Argo3
Ptgs1
rPL7
b
Dgcr8
Dicer
Xpo5
Argo2
Relative amounts
After E2 treatment (h)
After E2+P4 treatment (h)

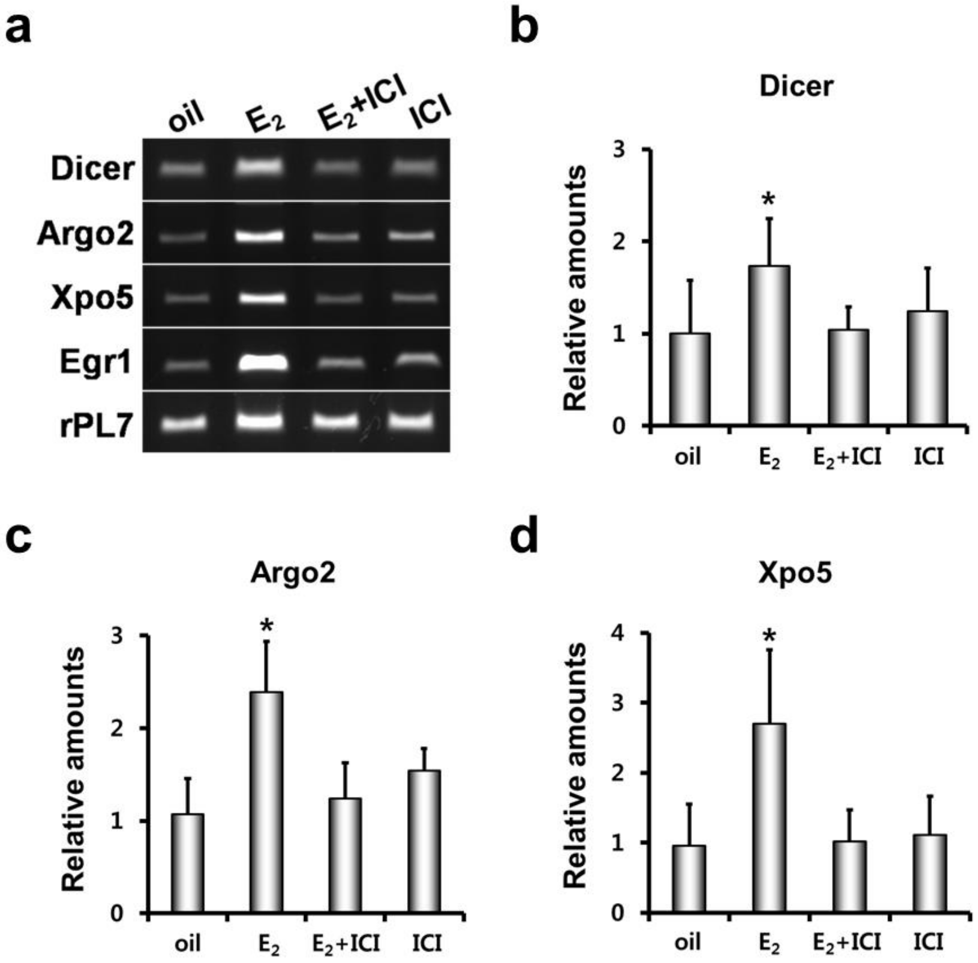

a
oil
E2
E2+ICI
ICI
Dicer
Argo2
Xpo5
Egr1
rPL7
b
Dicer
Relative amounts
0
1
2
3
*
oil
E2
E2+ICI
ICI
c
Argo2
Relative amounts
0
1
2
3
*
oil
E2
E2+ICI
ICI
d
Xpo5
Relative amounts
0
1
2
3
4
*
oil
E2
E2+ICI
ICI

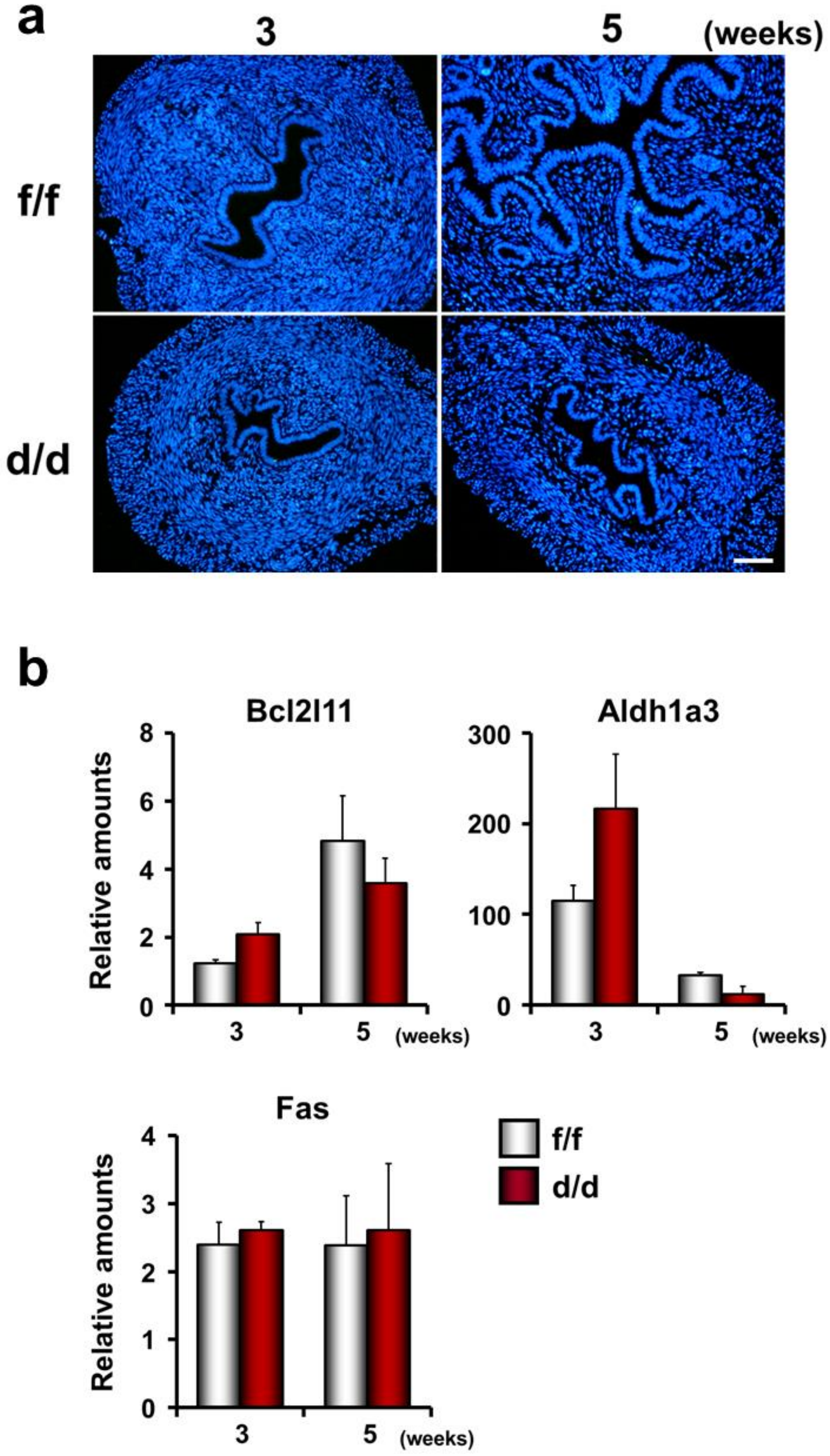
a
3
5
(weeks)
f/f
d/d
b
Bcl2l11
Aldh1a3
Fas
Relative amounts
8
6
4
2
0
300
200
100
0
4
3
2
1
0
3
5
(weeks)
f/f
d/d
